# Mechanical Properties of Robocast Glass Scaffolds Assessed through Micro-CT-Based Finite Element Models

**DOI:** 10.3390/ma15186344

**Published:** 2022-09-13

**Authors:** Luca D’Andrea, Dario Gastaldi, Enrica Verné, Francesco Baino, Jonathan Massera, Gissur Örlygsson, Pasquale Vena

**Affiliations:** 1 Laboratory of Biological Structure Mechanics (LaBS)—Politecnico di Milano, Department of Chemistry, Materials and Chemical Engineering Giulio Natta, Piazza Leonardo da Vinci 32, 20133 Milano, Italy; 2 Institute of Materials Physics and Engineering, Department of Applied Science and Technology—Politecnico di Torino, 10129 Torino, Italy; 3 Faculty of Medicine and Health Technology, Tampere University, 33100 Tampere, Finland; 4 IceTec, 112 Reykjavik, Iceland

**Keywords:** bioactive glass, scaffold, computed micro-tomography, strength, robocasting

## Abstract

In this study, the mechanical properties of two classes of robocast glass scaffolds are obtained through Computed micro-Tomography (micro-CT) based Finite Element Modeling (FEM) with the specific purpose to explicitly account for the geometrical defects introduced during manufacturing. Both classes demonstrate a fiber distribution along two perpendicular directions on parallel layers with a $90^{\circ }$ tilting between two adjacent layers. The crack pattern identified upon compression loading is consistent with that found in experimental studies available in literature. The finite element models have demonstrated that the effect of imperfections on elastic and strength properties may be substantial, depending on the specific type of defect identified in the scaffolds. In particular, micro-porosity, fiber length interruption and fiber detaching were found as key factors. The micro-pores act as stress concentrators promoting fracture initiation and propagation, while fiber detachment reduces the scaffold properties substantially along the direction perpendicular to the fiber plane.

## 1. Introduction

The goal of Bone Tissue Engineering (BTE) scaffolds is to repair critical size bone tissue defects by providing proper load-bearing capacity and promoting the mineralization process. As such, structural integrity in terms of stiffness and strength has to be provided so to guarantee biomechanical compatibility; furthermore, micro-architectural features should permit appropriate fluid flow and maximize osteo-integration [1].

In this paper, bioactive glasses are selected as biomaterials for scaffolding [2,3,4], capable of creating a stable tissue/device interaction [5,6]. However, the intrinsic low fracture toughness of glasses, especially in a highly porous devices, has limited their clinical application. Therefore, further material development as well as new manufacturing technologies and design strategies are being largely investigated [7]. The recent introduction of Additive Manufacturing (AM) techniques in BTE scaffold production have opened new horizons [8] towards the fine modulation of the morphological and mechanical properties of the scaffolds. In this work, the robocasting AM technology is considered; it is an extrusion-based printing technique [9]. The design of optimal architecture for a specific scaffold can be achieved through modern modeling and optimization techniques [10,11,12,13]. However, the manufacturing technologies unavoidably produce intrinsic microstructural defects such as micro-voids and micro-cracks. Furthermore, in case of the robocasting of glass scaffolds, thermal treatment will produce a deviation of the printed scaffold geometry from the designed micro-architecture. It is therefore of primary importance to assess the expected mechanical properties of real scaffolds through models allowing for the actual geometry and defect distribution of the manufactured devices.

To this specific purpose, Computed micro-Tomography (micro-CT) is the method of choice that makes the required information available. The estimate of the macroscopic elastic property of bone scaffold through micro-CT finite element modeling is relatively well established (see for example [14]); however, the estimate of the scaffold strength is still a challenging task. Miranda et al. [15] applied a traditional finite element approach to determine the compressive strength of robocast glass scaffolds, confining their study to ideal geometries. Entezari et al. [16], used the extended finite element method (XFEM), obtaining a satisfactory prediction of the scaffold strength and of the fracture pattern.

The strength of brittle-like materials can be predicted using probabilistic approaches; as an example, a two scale approach was used by Genet et al. [17] on robocast scaffolds. Unlike probabilistic approaches, the simulation of the fracture process can predict more accurately both the overall strength and the fracture pattern in a complex three-dimensional structure.

In [18], Farina et al. applied an iterative procedure to simulate the failure mechanisms in glass scaffolds, demonstrating how selected geometrical features affect the overall strength of the scaffolds.

The aim of this paper is to determine the elastic and strength properties of two sets of glass scaffolds obtained through robocasting, taking into account the actual distribution of material defects resulting from the manufacturing process. Once the macroscopic uniaxial elastic modulus and compressive strength are partially validated against experimental data, elastic moduli along all spatial directions and compressive strength along the three principal directions of the scaffolds are also obtained with the purpose to predict mechanical properties not obtained through the available experiments.

## 2. Materials and Methods

### 2.1. Scaffold Manufacturing, Micro-CT Scans and Defect Identification

Two sets of bioactive glass scaffolds, based on the compositional 47.5B silicate system and fabricated by robocasting according to previous studies [19,20], have been analyzed in this work: (i) three scaffolds exhibiting a gradient in the fiber spacing (graded) and (ii) three scaffolds exhibiting homogeneous fiber (monopore). The first set of scaffolds has been manufactured by printing fibers with lateral spacing between parallel fibers, which is increasing from the lateral part of the sample towards the center (fiber spacing decreased from 636μm in the external region to 51μm in the internal region); while the second set of samples is obtained by printing the fibers uniform spacing of 200μm.

The volumes of the scaffolds have been reconstructed starting from high resolution micro-CT images (pixel size 5μm).

A detailed explanation of the manufacturing process and the micro-CT image acquisition can be found in [18]; a complete experimental characterization of scaffolds, including in vitro bioactivity and in vivo bone-regenerative properties, is reported in [21].

Each set of micro-CT data contained a set of 16-bit grey-scale images. A gaussian filter with 2 pixel radius is first applied to reduce the noise. Then, thresholding procedure and binarization were applied to the whole set by using the Otsu algorithm [22].

The intrinsic porosity, due to bubbles in the slurry forming during the manufacturing process [23], has been quantified by measuring the size and shapes of the voids into the fibers. The intrinsic porosity was identified by inverting the binary images (void into solid and vice-versa) and analyzed through the 3D object counter built-in tool in Fiji, excluding the largest volume (corresponding to the interconnected major porosity). The intrinsic porosity has been computed as the ratio between the volume of the micro-voids within the fibres and the volume of the solid part. The shape of the micro-voids has been characterized, as follows. Let *a*, *b* and *c* be the three semi-axes of the fitting ellipsoid such that a>b>c: if a/c>1+δ and a/b>1+δ the ellipsoid is classified as prolate, if a/c>1+δ and 1−δ<a/b<1+δ the ellipsoid is oblate, otherwise the pore is classified as a spheroid. In this work, the parameter δ has been set to 0.4, as a representative threshold of 40% deviation from a spherical shape.

### 2.2. Micro-CT Based Finite Element Modeling

All finite element analyses were conducted by means of the open source multigrid finite element code ParOSol [24].

The 3D solid volume of each scaffold was cropped in a cuboid domain and imported in the finite element model by creating a cartesian mesh (voxel-type) in which each voxel corresponds to one single cubic element with 8 nodes, one node at each vertex.

The element size was 10μm in all finite element simulations. Depending on the solid volume fraction of the specific scaffold, the obtained finite element meshes contained approximately from 77 to 105 millions elements for the elastic analyses and from 34 to 40 millions elements for the strength analysis.

The elastic analyses were conducted on the largest domain cropped on the stack, having the size of 1200×1200×800 pixel; in order to save computational time, a resampling process of 2 pixel has been performed in the three cartesian directions obtaining a volume of 600×600×400 pixel, with the pixel size of 10μm. The simulated 3D domain size was 6mm×6mm×4mm, 24 times the fiber diameter, which is approximately 250μm.

Concerning the strength analyses, cubic domains have been cropped in the middle of the whole volume, with 400 pixel per edge and a pixel size of 10μm, which corresponds to a 3D domain of 4mm×4mm×4mm, 16 times the fiber diameter.

#### 2.2.1. Elastic Analyses

In order to determine the full elastic tensor at the macroscopic scale, the representative volume element was used in six different finite element analyses; each analysis was carried out by applying displacement boundary conditions macroscopically equivalent to a unit uniaxial strain. If the six independent macroscopic strain components are listed in a six-entries array, the six macroscopic strain states are those reported in Equation (1):

(1) $$\varepsilon^{1}=\left[\begin{array}{c} 1\\ 0\\ 0\\ 0\\ 0\\ 0 \end{array}\right];\varepsilon^{2}=\left[\begin{array}{c} 0\\ 1\\ 0\\ 0\\ 0\\ 0 \end{array}\right];\varepsilon^{3}=\left[\begin{array}{c} 0\\ 0\\ 1\\ 0\\ 0\\ 0 \end{array}\right];\varepsilon^{4}=\left[\begin{array}{c} 0\\ 0\\ 0\\ 1\\ 0\\ 0 \end{array}\right];\varepsilon^{5}=\left[\begin{array}{c} 0\\ 0\\ 0\\ 0\\ 1\\ 0 \end{array}\right];\varepsilon^{6}=\left[\begin{array}{c} 0\\ 0\\ 0\\ 0\\ 0\\ 1 \end{array}\right];$$

where the first three entries represent direct strain component, while the last three entries represent the shear strain components. Uniform kinematic boundary conditions were used, instead of periodic boundary conditions, as the printed structures do not present a perfect periodicity. The size of the domain, with respect to the fiber diameter, was deemed to be large enough to have a limited overestimation of the elastic modulus, resulting in uniform kinematic boundary conditions on small domains.

A unit elastic modulus (E=1 MPa) and a Poisson ratio ν=0.3 have been assumed for the solid phase to obtain the normalized macroscopic elastic modulus. The real elastic properties of the scaffolds have been obtained by multiplying the normalized properties by the elastic modulus of the glass material.

The entries of the 6×6 elastic stiffness matrix L at the macroscopic scale have been obtained by averaging the micro-level stress components, as conducted in [25]:

(2) $$L_{ij}=\frac{1}{V}\int_{V}\sigma_{j}^{i}dV$$

in (2), the microscopic-scale level stress tensor components have been arranged in a six-entries vector following the same ordering as that defined for the strain tensor components; $\sigma_{j}^{i}$ is the $j^{th}$ entry of the stress vector for the $i^{th}$ set of boundary conditions, as defined in (1).

In order to be rid of numerical errors, the resulting stiffness matrix was symmetrized by taking the symmetric part of L.

In order to determine the direction dependent elastic modulus, E(α,ϕ) is obtained, being α and ϕ the azimutal and elevation angles, respectively, which identify the spatial direction with respect to the orthogonal cartesian reference system having the axes aligned with the principal directions of the scaffolds. The E(α,ϕ) is obtained, as reported in (3):

(3) $$E{\left(\alpha ,\phi \right)}^{-1}=n_{i}n_{j}n_{k}n_{l}D_{ijkl}$$

in which $D_{ijkl}$ is the fourth order compliance tensor obtained through the inverse of the elastic stiffness matrix and n is the unit vector, which identifies the spatial directions through the angles α,ϕ:

(4) $$n^{T}=\left[cos(\phi )cos(\alpha )\;\;cos(\phi )sin(\alpha )\;\;sin(\phi )\right]$$

The macroscopic elastic modulus along the direction perpendicular to the printing plane will be $E(\alpha ,\frac{\pi }{2}),\forall \theta$; while the elastic modulus along the two directions of the fiber printing will be E(0,0) and $E(\frac{\pi }{2},0)$.

#### 2.2.2. Assessment of Strength

The macroscopic strength was assessed by running a finite element simulation of a uniaxial compression test. Two load directions have been simulated, namely the z-direction (perpendicular to both families of fibers) and x-direction (parallel to a family of fibers). For a compression along the z-direction $u_{z}=0$ is enforced on the boundary with outward normal (0,0,−1); while for a compression along the x-direction, $u_{x}=0$ is enforced on the boundary with outer normal (−1,0,0). The opposite faces were subjected to displacement perpendicular to the face so to simulate a progressive displacement-controlled compression test.

The lateral boundaries of the domain were subjected to stress-free conditions. Rigid body motions were constrained by subjecting two non parallel faces of the external lateral surface to a symmetry condition. The procedure described in [18] was used to simulate the mechanical response of the scaffold until first significant drop of macroscopic stress was detected. Briefly, the procedure is a sequence of linear analyses with check on the material strength on all elements at each step. The strength criterion used to simulate fracture process of the glass material was the Drucker-Prager criterion, which allows for a tension-compression mismatch in material strength. The fracture propagation process was simulated by removing, at each sequential linear analysis step, a prescribed number of elements among the most stressed ones; the corresponding value of the applied displacement on the boundary is found so to ensure that all the remaining elements fulfill the Drucker-Prager strength criterion. A sensitivity analysis on the selection of the number of elements to be removed at each analysis step is presented in [18].

The parameters of the Drucker-Prager criterion were calculated by setting a tensile strength of 47 MPa. According to the results found for many ceramic materials [26], the compressive strength was set to 10 times the tensile strength. The remaining of the parameters for the sequential procedure were assumed as in the work of Farina et al. [18].

## 3. Results

The total porosity of the graded and monoporous scaffolds is 44±2% and 32±8%, respectively, whereas the intrinsic porosity corresponds to 0.39±0.45% and 0.60±0.10%, respectively. The shape of the micro-voids constituting the intrinsic porosity is mostly a prolate ellipsoid (70% of the total number of particles) mainly aligned with the fiber axis. This particular shape lets us speculate that these are the results of air bubbles entrapped during the extrusion process. The dimensions of the semi-axes of the fitting ellipsoids with an equivalent radius greater than 30μm are 82.67±19.37μm, 37.25±6.74μm and 25.35±5.68μm.

**Figure 1:**
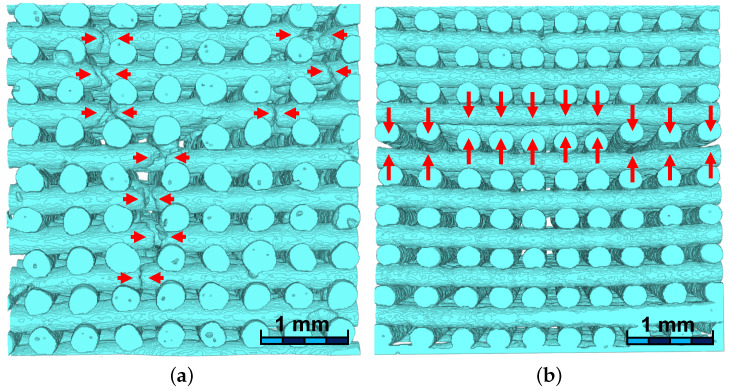
Micro-CT reconstruction of two scaffolds. Geometric imperfections are described: (**a**) fibers interruption identified by red arrows and (**b**) fibers detaching between two adjacent printing planes.

In addition to the micro-porosity found within the fibers, two different types of geometrical imperfections of the structure have been found (see red arrows in Figure 1): (i) the interruption of the fibers along their longitudinal axes (panel (a)) and, (ii) a non connecting region between two adjacent printing planes (panel (b)), i.e., fibers are detached from their resting plane.

While the micro-porosity within the fibers was found in both robocast sample groups, the two geometrical imperfections were found in the graded samples only.

The macroscopic Young’s moduli and shear moduli are reported in Table 1 as normalized values with respect to the elastic modulus of the solid material. In the table, standard deviation is also reported on the three samples analyzed for each group.

**Figure 2:**
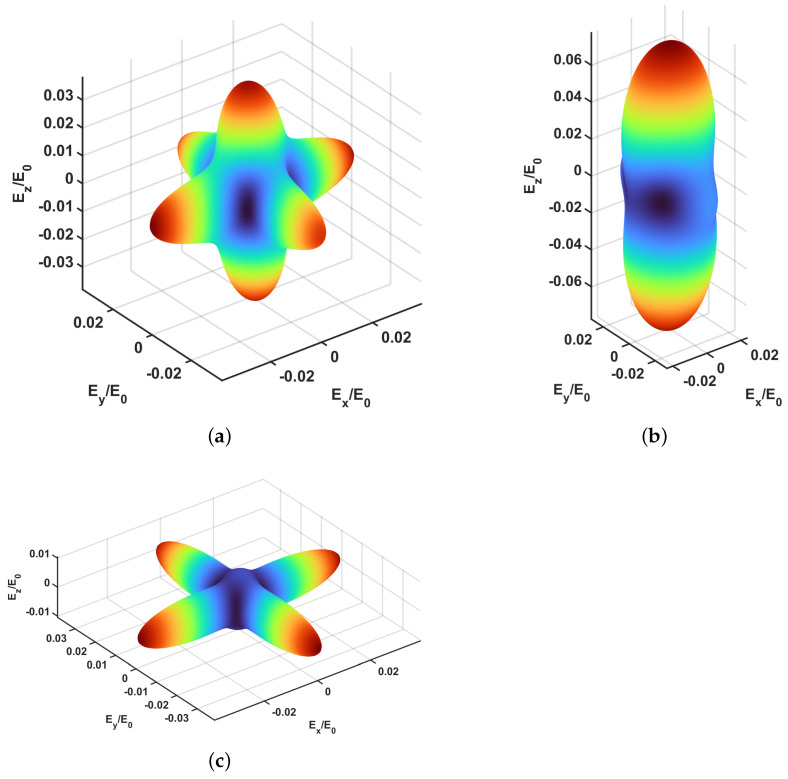
3D representation of the macroscopic stiffness of the scaffolds: (**a**) cubic symmetry scaffolds, (**b**) low porosity scaffolds and (**c**) scaffolds exhibiting detaching of fibers.

Figure 2 shows the normalized elastic modulus along all spatial directions. Although the six scaffolds have been grouped in two families, namely graded and monoporous, the numerical results suggest three different mechanical responses: (i) samples exhibiting cubic symmetry (panel (a)), (ii) samples with low porosity, exhibiting an elastic modulus along the fibers substantially smaller than that along the vertical direction (panel (b)) and, (iii) samples characterized by fibers detaching defect (panel (c)), which is characterized by a substantially lower elastic modulus along the z-direction.

**Figure 3:**
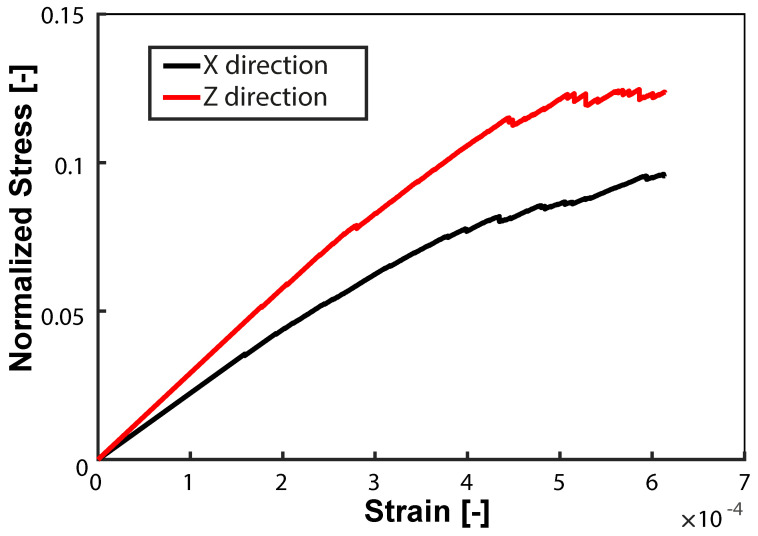
Stress–strain curves under uniaxial load along two different directions.

The macroscopic strength of the two classes of scaffolds are reported in Table 2. Since the scaffolds exhibit a quasi-brittle behavior, the highest peak of the stress-strain response has been considered as the strength of the scaffolds. Figure 3 is a representative example of the macroscopic stress versus macroscopic strain curves upon the unconfined compression along the x-direction (parallel to the fibers) and along the z-direction (perpendicular to the fibers’ printing plane). Two of the graded samples exhibited a low normalized strength (0.01±0.004), while the third graded sample, as well as the monoporous samples, exhibited a normalized strength, which closely followed a third order polynomial fitting, as conducted in [18].

## 4. Discussion

The graded samples exhibit an elastic modulus along the z-direction which is, on average, approximately 66% lower than that of the monoporous samples ($E_{z}/E_{0}=0.44$ versus $E_{z}/E_{0}=0.15$). This reduction is owed to two phenomena: (i) the graded scaffolds exhibit higher overall porosity, which negatively affects the elastic modulus and (ii) graded scaffolds exhibit the geometrical imperfections, as described in the method section. If scaffolds featuring the aforementioned fibers detaching are excluded, both sets of scaffolds follow a cubic stiffness-porosity relationship, as reported by Farina et al. [18].

For both classes of scaffolds, no statistically significant differences have been found for the normalized stiffness along the two directions parallel to the fibers, namely $E_{x}/E_{0}$ and $E_{y}/E_{0}$. The mismatch in $E_{x}/E_{0}$ (or $E_{y}/E_{0}$) between monoporous and graded samples is lower (0.28 versus 0.18) with a reduction of 36% in the graded samples. Both classes of scaffolds demonstrated a shear modulus $G_{xy}$ lower than the shear moduli along the two other planes ($G_{yz}$ and $G_{xz}$): this behavior is consistent with the fabric-like geometry of the scaffold in the xy-plane. Furthermore, graded samples also exhibit, on average, a shear stiffness lower than that demonstrated by the monoporous samples ($G_{xy}/E_{0}=0.065$ versus $G_{xy}/E_{0}=0.031$). It can be speculated that the simultaneous reduction of elastic modulus along the z-direction and of the shear stiffness in the xy-plane is an indication of a smaller overlap of fibers on two subsequent printing planes. Figure 1b shows a micro-CT reconstruction of one of the graded samples exhibiting the lowest elastic modulus along the z-direction. A geometrical imperfection is clearly visible where detached fibers have been found. The detachment of fibers is responsible for a substantial decrease in the elastic modulus along the z-direction, whereas it does not affect the elastic moduli along the two other directions, where the continuity of the fibers is preserved.

**Figure 4:**
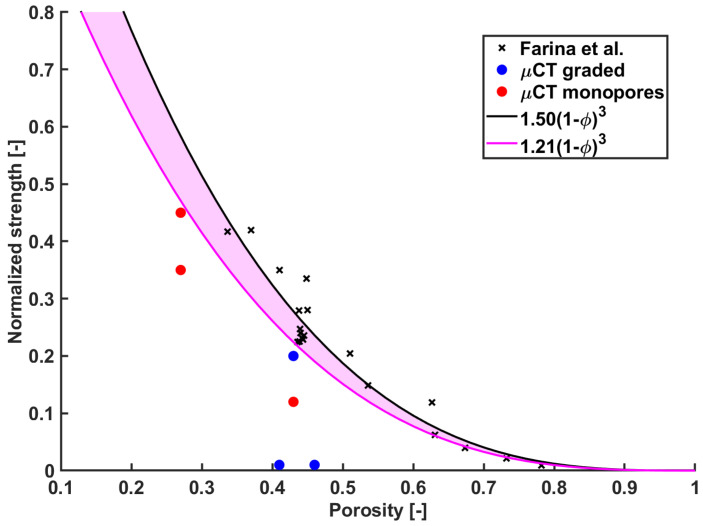
Best fitting curve of ideal scaffolds (black line) from Farina et al. and best fitting curve also considering real scaffolds (purple line).

Figure 4 shows the normalized strengths of all samples, along with the samples having idealized geometry without intrinsic porosity. If a fitting function $\frac{\bar{\sigma }}{\sigma_{0}}=A{\left(1-\phi \right)}^{3}$ is used, the fitting constant *A* found in this study is 1.21, while A=1.5 was found by Farina et al. [18]. The higher value of *A* in [18] is owed to the fact that scaffolds with idealized geometry and without intrinsic porosity were included in the dataset for fitting.

Barberi et al. [23] performed compression tests on robocast samples having the same geometry, as the analyses in this paper with an overall porosity ϕ=49.1±5.5% and found a macroscopic compressive strength of σ=6.1±2.6MPa. The normalized strength found in our study at the same porosity can be estimated by using the cubic fitting curve as $\frac{\sigma }{\sigma_{0}}=1.21{\left(1-\phi \right)}^{3}{|}_{\phi =0.49}=0.16$. If the experimental strength σ=6.1 MPa is used, the resulting intrinsic material strength is $\sigma_{0}=\frac{6.1}{0.16}=38$ MPa. It is worth noticing that since the polynomial best fitting between the porosity and the strength has been obtained combining ideal and real scaffolds, the computed intrinsic strength may slightly under-estimate the intrinsic material strength.

The macroscopic strength along z-direction, obtained with our finite element models of monoporous and graded samples with intrinsic material strength $\sigma_{0}=38\;\mathrm{MPa}$ is 2.88±3.51 MPa and 11.79±5.22 MPa, respectively; while the strength along the x-direction is 9.38±1.99 MPa and 2.90±0.84 MPa.

**Figure 5:**
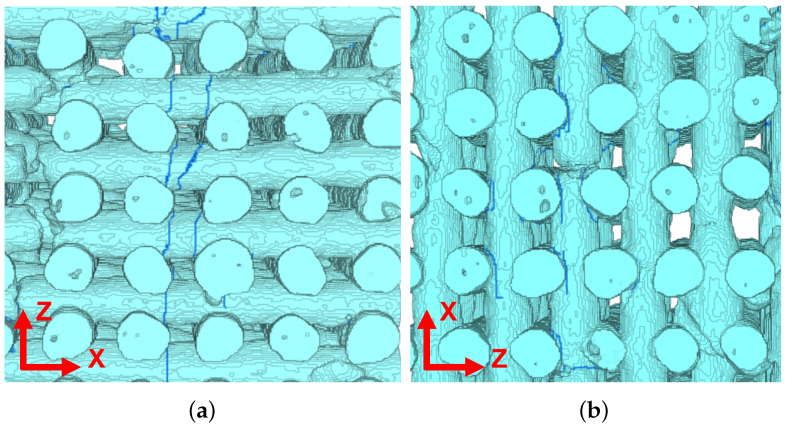
Crack pattern upon compressive load: (**a**) the load is orthogonal to both families of fibers and (**b**) the load is directed along the x-fibers.

Figure 5 shows the crack pattern found after compression along z and x directions in the left and right panels, respectively. As a tension/compression mismatch is used, the fracture mainly occurred in regions subjected to tensile stress. The figure shows that the compression perpendicular to the fiber planes causes fractures surfaces, which are perpendicular to the fiber axis, and mostly close to the fiber intersections. The compression along the fibers produces fractures surfaces, which are parallel to the fiber (aligned with the loading direction). The latter are mostly located at the fiber intersections. Both failure patterns of the above described are consistent with that experimentally found by Miranda et al. (compare Figure 5 with Figures 5 and 8 in [27] and with Figure 12e in [28]). The compression along the fiber direction eventually brings fiber rotations, as highlighted in [27]. This particular failure mode cannot be caught by the finite element model presented in this paper, as linear kinematics is assumed and fiber rotation after cracking can be obtained only if finite displacements are modeled.

**Figure 6:**
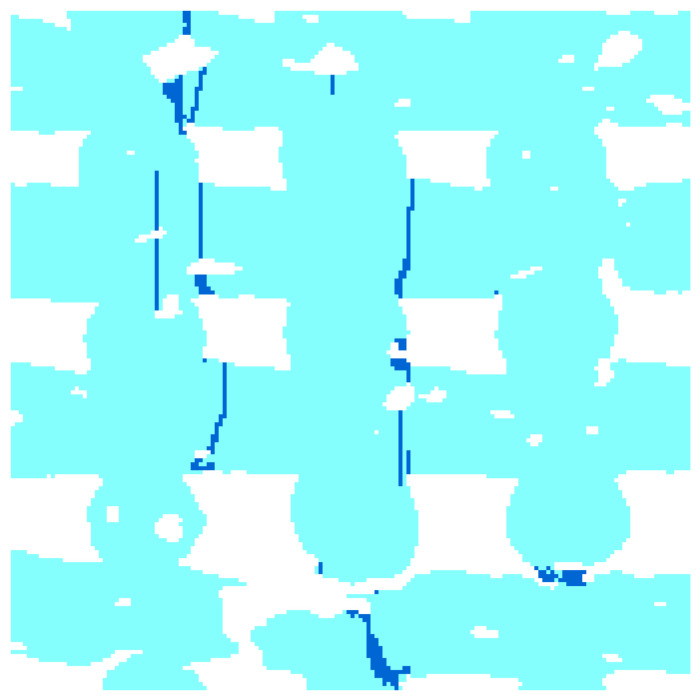
Section of the scaffold in the xz-plane, under compressive load along the z-direction (vertical). Cracks are highlighted in dark blue.

The crack pattern revealed in the finite element simulations of compressive load along directions perpendicular to the fibers is characterized by fracture surfaces mainly perpendicular to the fiber longitudinal direction. As substantial tensile/compressive strength mismatch is assumed in the numerical simulation, tensile stress is the major responsible factor for cracking, suggesting that mode I crack opening is predominant [27]. Figure 6 shows that, when intrinsic microporosity occurs within the fibers, the cracks initiate or cross the pores due to stress concentration at the pore boundary.

**Figure 7:**
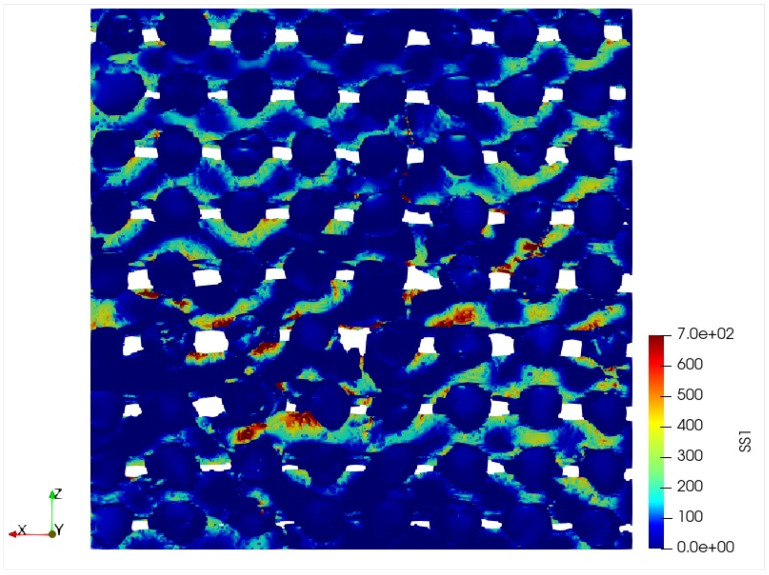
Color plot of stress component $\sigma_{xx}$ upon compression along z-direction.

Figure 7 shows a color plot of the $\sigma_{xx}$ stress component for a scaffold subjected to compression along the z-direction (vertical direction in the figure). The tensile stresses are highlighted, showing that the compression along the vertical direction can cause tensile stress along the horizontal fibers; depending on the local geometrical shapes of the fibers, high values of tensile stress are also found at the center of the free segment between the two adjacent fibers running along the y-direction (perpendicular to the plane of the figure). This finding is consistent with numerical results reported in [27].

Petit et al. [28] conducted a similar study, in which the effects of micro-voids on robocast samples are simulated by introducing spherical cavities into the model. Their finite element grid exhibited an element characteristic size of 69μm and, therefore, defects smaller than that size could not be accounted for. In the present work, the element size is 10μm; this allowed us to assess the role played by smaller defects, which are of practical interest for this specific manufacturing technology.

This work is mainly limited by the small number of samples on which computational analyses have been performed due to the limited availability of computed tomography data. In addition to the limitations of the computational procedure already discussed in [18], the crack pattern presented in this work, although consistent with the experimental data presented by others, is affected by the voxel-type nature of the finite element mesh, which makes the fracture paths to feature straight and jigsaw shapes. This is an un-natural fracture surface; however, the high spatial resolution of the geometry (i.e., the small size of the finite elements) makes the crack patterns reasonable and able to identify the failure mechanisms.

One of the early use of finite element modeling based on a voxel-mesh was in the field of homogeneization technique for cellular materials as trabecular bone [29] in the elastic range, or in topology optimization [30,31]. Unlike elasticity, the use of voxel-mesh for strength analyses needs particular care [32]. Considering this, sensitivity analyses on model parameters used in this paper were performed in [18]. Furthermore, in this work, an independent measure of the intrinsic tensile strength of the solid material is missing. To the the authors’ knowledge, experimental data on intrinsic strength of the solid glass material as obtained through the robocasting technique are still missing. In [33], the tensile strength of silicate glass (SCNA) is estimated to be 47 MPa, which is very close to the estimation found in this work for the 47.5B-based silica glass; however, as chemical composition and manufacturing technology are different, comparison should be taken with care. Indeed, suitable experiments would be needed in order to corroborate the assessment of this property. In particular, micro-tensile or micro-bending tests would be needed on bulk samples having the characteristic size of the fiber diameter, manufactured using the same extrusion technique and the same thermal treatments. This would allow one to determine the intrinsic mechanical properties of the material, getting rid of the effects induced by the intrinsic geometrical imperfections of the grid-like geometry of the scaffolds.

## 5. Conclusions

In this work, micro-CT finite element analyses were performed with the purpose to determine the macroscopic elastic properties of robocast glass bone tissue engineering scaffolds. Furthermore, the compressive strength along the three cartesian directions of the scaffolds was investigated by means of an approximate incremental procedure simulating the local material failure by sequential finite element analyses based on micro-CT immages [18]. In particular, the role of the geometrical imperfections of the scaffolds is discussed. Three different types of microscopic defects were identified through the micro-CT image analyses, namely micro-porosity in the extruded fibers, fiber detachment and fiber interruptions. These defects have a substantial effect on the overall mechanical properties and on the crack pattern obtained in the simulated compression tests. The satisfactory comparison with available data in literature, in terms of fracture pattern, makes the proposed model a valuable computational framework to be used as a predictive tool for the design of reliable robocast glass bone scaffolds.

## Figures and Tables

**Table 1:** Dimensionless macroscopic Young’s moduli and shear moduli for both classes of scaffolds.

	$E_{x}/E_{0}$	$E_{y}/E_{0}$	$E_{z}/E_{0}$	$G_{\mathrm{xy}}/E_{0}$	$G_{\mathrm{yz}}/E_{0}$	$G_{\mathrm{xz}}/E_{0}$
graded	0.28 ± 0.02	0.27 ± 0.03	0.15 ± 0.08	0.03 ± 0.01	0.06 ± 0.01	0.06 ± 0.02
monoporous	0.19 ± 0.02	0.17 ± 0.04	0.44 ± 0.14	0.07 ± 0.02	0.11 ± 0.03	0.11 ± 0.03

**Table 2:** Dimensionless macroscopic strength for both classes of scaffolds.

	$\sigma_{x}$	$\sigma_{z}$
graded	0.24 ± 0.05	0.08 ± 0.09
monoporous	0.08 ± 0.02	0.31 ± 0.14

## Data Availability

Not applicable.
